# β-Lapachone Selectively Kills Hepatocellular Carcinoma Cells by Targeting NQO1 to Induce Extensive DNA Damage and PARP1 Hyperactivation

**DOI:** 10.3389/fonc.2021.747282

**Published:** 2021-10-05

**Authors:** Wenxiu Zhao, Lingxiang Jiang, Ting Fang, Fei Fang, Yingchun Liu, Ye Zhao, Yuting You, Hao Zhou, Xiaolin Su, Jiangwei Wang, Sheng Liu, Yaomin Chen, Jun Wan, Xiumei Huang

**Affiliations:** ^1^ Department of Radiation Oncology, Melvin and Bren Simon Comprehensive Cancer Center, Indiana University School of Medicine, Indianapolis, IN, United States; ^2^ Fujian Provincial Key Laboratory of Chronic Liver Disease and Hepatocellular Carcinoma, Zhongshan Hospital, Xiamen University, Xiamen, China; ^3^ Departments of Biochemistry and Molecular Biology, Melvin and Bren Simon Comprehensive Cancer Center, Indiana University School of Medicine, Indianapolis, IN, United States; ^4^ Department of Medical and Molecular Genetics, Indiana University School of Medicine, Indianapolis, IN, United States; ^5^ Indiana University Health Pathology Laboratory, Indiana University School of Medicine, Indianapolis, IN, United States; ^6^ Center for Computational Biology and Bioinformatics, Indiana University, School of Medicine, Indianapolis, IN, United States

**Keywords:** beta-lapachone, NQO1, hepatocellular carcinoma, DNA damage/reactive oxygen species, NAD^+^/ATP depletion

## Abstract

Hepatocellular carcinoma (HCC) is the second leading cause of cancer-related death globally. Currently there is a lack of tumor-selective and efficacious therapies for hepatocellular carcinoma. β-Lapachone (ARQ761 in clinical form) selectively kill NADPH: quinone oxidoreductase 1 (NQO1)-overexpressing cancer cells. However, the effect of β-Lapachone on HCC is virtually unknown. In this study, we found that relatively high NQO1 and low catalase levels were observed in both clinical specimens collected from HCC patients and HCC tumors from the TCGA database. β-Lapachone treatment induced NQO1-selective killing of HCC cells and caused ROS formation and PARP1 hyperactivation, resulting in a significant decrease in NAD^+^ and ATP levels and a dramatic increase in double-strand break (DSB) lesions over time *in vitro*. Administration of β-Lapachone significantly inhibited tumor growth and prolonged survival in a mouse xenograft model *in vivo*. Our data suggest that NQO1 is an ideal potential biomarker, and relatively high *NQO1:CAT* ratios in HCC tumors but low ratios in normal tissues offer an optimal therapeutic window to use β-Lapachone. This study provides novel preclinical evidence for β-Lapachone as a new promising chemotherapeutic agent for use in NQO1-positive HCC patients.

## Introduction

As the most common primary liver cancer and the second leading cause of cancer-related deaths worldwide (1), hepatocellular carcinoma (HCC) patients are usually diagnosed with advanced disease, resulting in only 15% of HCC patients being eligible for surgical resection or liver transplantation and the median survival time for HCC patients in intermediate to advanced stages being only 1-2 years (2). Moreover, chemotherapy against HCC has limited benefits because of the high resistance to currently available chemotherapeutic agents. Sorafenib, the first-line drug used for patients with advanced HCC, has been used for over 10 years, but the overall outcomes are unsatisfactory (3). These have led to more extensive research focusing on personalized medicine with increased selectivity and efficacy.

A growing body of data demonstrates that NADPH:quinone oxidoreductase 1 (NQO1), a phase II two-electron reductase that can bioactivate certain quinone molecules and shows a protective effect against natural and exogenous quinones, is abnormally upregulated in many solid cancers, such as lung, pancreatic, breast, prostate, and colon cancers (4–11). In liver cancer, it has been reported that NQO1 was increased 18-fold in HCC versus normal livers (12). Recently, NQO1 overexpression was reported to be a potent independent biomarker for prognostic evaluation of HCC (13) and enhanced apoptosis inhibition of liver cancer cells *via* the SIRT6/AKT/XIAP signaling pathway (14, 15). NQO1 overexpression in tumors has the advantage of preferentially killing cancer cells and sparing normal cells when anticancer drugs that are bioreductively activated by NQO1, such as β-Lapachone (β-lap), are used (5).

β-lap has gained increasing attention for its tumor-selective and antitumor effects in many cancers, including lung cancer, breast cancer, prostate cancer, pancreatic cancer, and leukemia (4–7, 9–11, 16, 17). Its toxicity is closely correlated with NQO1 expression and activity. Our studies suggest that NQO1 metabolizes β-lap through a futile redox cycle in which β-lap is converted into a highly unstable hydroquinone form and then spontaneously reacts with oxygen to revert back to the parent compound, causing rapid NAD(P)H oxidation. This process generates high levels of reactive oxygen species (ROS) (e.g., H_2_O_2_), resulting in genomic instability and DNA damage (6, 11). In addition, catalase (*CAT*) can bypass β-lap toxicity by neutralizing hydrogen peroxide produced by β-lap (18). We have previously reported that β-lap alone or combined with other inhibitors had profound toxicity in pancreatic cancer and non-small-cell lung cancer (NSCLC) (11, 19–21). However, the effect of β-lap on HCC is virtually unknown.

Here, we demonstrate that HCC patient samples have significantly elevated levels of NQO1 but concomitantly low catalase levels compared with associated normal tissues. HCC cells were efficiently killed by β-lap, along with increases in ROS production and PARP1 hyperactivation, severe NAD^+^/ATP depletion and DNA damage. NQO1-dependent HCC killing was confirmed in HCC cells with stable NQO1 overexpression and knockout. Furthermore, a human HCC subcutaneous xenograft mouse model exhibited efficient β-lap-induced control of tumor growth and prolonged mouse survival.

## Materials and Methods

### Human HCC Cell Lines and Clinical Samples

Human HCC cell lines (SNU-182, PLC/PRF/5, Huh7, Hep3B, HepG2, and Li7) were purchased from Guangzhou Cellcook Biotechnology Co., Ltd. (Guangzhou, China), and PLC/PRF/5 and SK-HEP1 cells were purchased from ATCC. The authentication of these cell lines was performed *via* comparisons with the STR database. Cells were cultured in Dulbecco’s modified Eagle’s medium (DMEM) supplemented with 10% fetal bovine serum (FBS; HyClone), 100 U/ml penicillin, and 100 U/ml streptomycin at 37°C and 5% CO_2_.

### Reagents and Chemicals

β-lap, synthesized by Dr. Bill Bornmann (M.D. Anderson, Houston, TX) was dissolved in DMSO for *in vitro* experiments or 20% HPβCD for *in vivo* experiments, and the concentrations were verified by spectrophotometry. Hydrogen peroxide (H_2_O_2_) and dicoumarol (DIC) were purchased from Sigma-Aldrich. HPβCD (>98% purity) was purchased from Cydodextrin Technologies Development, Inc. The ROS-Glo™ H_2_O_2_ assay kit, NAD/NADH-Glo kit, and CellTiter-Glo^®^ 2.0 kit for the ATP assay were obtained from Promega Corporation. An alkaline comet assay kit was purchased from Trevigen, Inc.

### NQO1 Knockout/Knockin Cells

CRISP/Cas9 NOQ1 knockout PLC/PRF/5 cells and NQO1-overexpressing SK-HEP1 cells were generated by our lab. Vectors of guide RNA sensing human NQO1 or nontarget control (LV04) and Cas9 expression (CAS9NEO) were provided by Sigma-Aldrich, and the guide RNA targeting sequences were AGGATACTGAAAGTTCGCAGGG and CACAATATCTGGGCTCAGATGG. Vectors with NQO1 or empty control (EX-Z0563-Lv205, EX-NEG-Lv205) were purchased from Sigma-Aldrich, and transfection of SK-HEP1 with these vectors was performed with Lipofectamine 3000 reagent (Thermo Fisher) according to the manufacturer’s protocol.

### Cell Survival Assay

A total of 10,000 cells/well were seeded on 48-well plates 24 h prior to treatment. Varying doses of β-lap dissolved in DMSO ± DIC were added and incubated for 2 h at 37°C and 5% CO_2_. After treatment, the media were replaced with fresh complete media and allowed to grow for 7 days. After 7 days, the cells were washed with 1x PBS, 200 μl of H_2_O was added, and the cells were frozen at -80°C for at least 2 h. After thawing, 200 μl/well TNE buffer (50 mM Tris–HCl (pH 7.4), 100 mM NaCl, 0.1 mM EDTA) with 1 μg/ml Hoechst 33258 was added and incubated for 1 h at room temperature in the dark. Cell growth was determined by absorbance at 560 nM with a multilabel plate reader (PerkinElmer). Percentage of cell growth = (100× (cell experimental – blank)): (cell control).

### Antibodies

Antibodies used for immunofluorescence and western blotting included NQO1 (A180, Santa Cruz, La Jolla, CA), PARP1 (F-2, Santa Cruz), β-actin (C4, Santa Cruz), α-tubulin (B-7, Santa Cruz), PAR (Trevigen, Gaithersburg, MD), γH2AX (JBW301, Millipore, Temecula, CA), H2AX (938CT5.1.1, Cell Signaling, Danvers, MA), and catalase (12980S, Cell Signaling, MA).

### Western Blotting Analysis

Cells were seeded on plates at approximately 70% confluence 24 h in advance and then treated with/without β-lap for 2 h. Next, the cells were lysed in lysis buffer. Approximately 40 μg of protein was resolved by SDS-PAGE, transferred onto PVDF membranes, and probed with antibodies. The protein-antibody complexes were detected by using Super Signal West Femto Substrate (Thermo Fisher, Waltham, MA) and exposure to film.

### Real-Time PCR

Assays were performed as previously described (22). The primer sequences were as follows: GAPDH-sense: 5’-CTGCTGATGCCCCCATGTTC-3’; GAPDH- antisense: 5’-CATCCACAGTCTTCTGGGTGG-3’; NQO1-sense: 5’-GCCATGTATGACAAAGGA CCC-3’; NQO1-antisense: 5’-ACTTGGAAGCCACAGAAATGC-3’; CAT-sense: 5’-CTTCGACCCAAGCAACATGC-3’; CAT-antisense: 5’-GCGGTGAGTGTCAGGATA GG-3’. 2^-ΔΔCt^ was used to calculate the fold change of mRNA expression.

### NQO1 and Catalase Activity Assays

Extracts were obtained from different HCC cell lines. Then NQO1 and catalase enzyme activities were assayed by NQO1 Activity Assay (Abcam) and CheKine™ catalase Activity Assay Kit (Abbkine), respectively, according to the manufacturer’s manual.

### ATP, NAD^+^ and Hydrogen Peroxide (H_2_O_2_) Assays

Cells were cultured (1 × 10^4^ cells/well) 24 h in advance in 96-well white-walled clear-bottom tissue culture plates (Sigma) and treated with β-lap with or without DIC for 2 h. Then, ATP (CellTiter-Glo), hydrogen peroxide (H_2_O_2_) (ROS-Glo), and NAD/NADH (NAD/NADH-Glo) were assayed at the indicated time points after treatments using specific assays (Promega).

### Comet Assay

Total DNA damage was measured by the alkaline comet assay (Trevigen) according to the manufacturer’s manual. Slides were stained with SYBR green, and images were acquired with a Leica DM5500 fluorescence microscope. Comet tail lengths were quantified by NIH ImageJ.

### Immunofluorescence Staining of γH2AX

The treated cells were fixed with 4% paraformaldehyde for 30 min, permeabilized with 0.2% Triton-X 100 for 10 min at 4°C, blocked with 3% bovine serum albumin for 30 min at room temperature, and incubated overnight at 4°C with γH2AX antibody (diluted at 1:1000). Cells were washed 3 times for 5 min in PBS and then incubated for 2 h with AlexaFluor secondary antibody (diluted 1:1000 in blocking buffer). DAPI was used to stain nuclei. γH2AX foci were visualized with a laser scanning confocal microscope (LSM 510 Meta), and the number of H2AX foci per nucleus was quantified.

### *In Vivo* Antitumor Study

All animal procedures were approved by the IU IACUC committee. For the *in vivo* xenograft model, PLC/PRF/5 cells (5×10^6^) were subcutaneously inoculated into the right flank of nonobese diabetic/severe combined immunodeficiency (NOD/SCID) male mice (6~8 weeks old). Tumor volumes were measured with a caliper and calculated by the formula 0.5×length×width^2^. When tumor volumes reached ~150 mm^3^, mice were randomly divided into vehicle (n=7) and treatment (n=8) groups with no significant differences in tumor sizes. Then, the mice were treated with HPβCD or HPβCD-β-lap (12.5 mg/kg) by intratumor injection every other day for a total of five injections. When the tumor volume reached ~1200 mm^3^, the mice were sacrificed, and a survival curve was plotted.

### Bioinformatic Analysis

The liver hepatocellular carcinoma (LIHC) dataset was downloaded from the Broad Institute TCGA Genome Data Analysis Center, https://doi.org/10.7908/C11G0KM9. Only samples with both tumor and matched normal samples were selected for further analysis of *NQO1* and *CAT* levels. The differences in gene expression levels for individual genes or fold changes of two genes between normal and tumor tissues were identified by paired t test.

### Statistical Analysis

All the experimental results were analyzed using two-tailed Student’s *t* tests for independent measures with Holm-Sidak correction for multiple comparisons if >1 comparison was performed. The minimum replicate size for experiment was *n*=3. Statistical analysis were performed in GraphPad Prism 8 (GraphPad Software, Inc. CA, USA). Images are representative of the results of experiments or staining repeated 3 times. Data are presented as the mean ± SD. The survival rate was analyzed by Kaplan-Meier survival curves. A p value of < 0.05 was considered statistically significant between the compared groups. *p < 0.05; **p < 0.01 and ***p < 0.001.

## Results

### High Expression of NQO1 in Hepatocellular Carcinoma Patients

**Figure 1:**
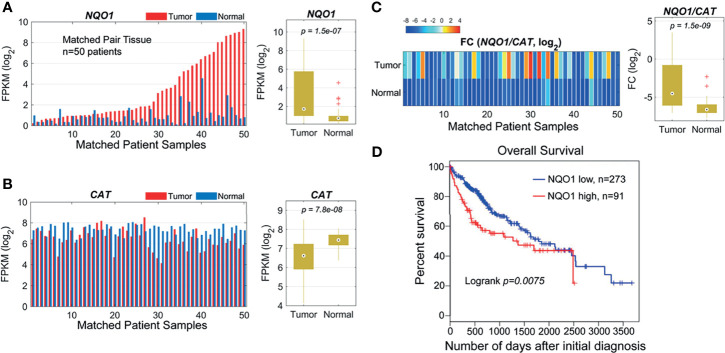
NQO1 and CAT expression profile in matched hepatocellular carcinoma patient samples (n = 50) in the TCGA cohort. **(A)** Left panel: NQO1 mRNA levels (FPKM, in log_2_ scale) in paired HCC tumor and normal liver tissues. The samples were sorted by NQO1 expression levels in tumors. Right panel: Distributions of FPKMs of NQO1 in liver tumor and normal tissues. **(B)** CAT gene expression in tumor and normal tissues (left panel) and FPKM distributions (right panel). **(C)** Left panel: The gene expression difference between two genes, NQO1 and CAT (fold change, FC, in log_2_ scale), for tumor and normal tissues, respectively. Right panel: Distributions of FCs in tumor and normal tissues. The orders of samples in **(B, C)** were exactly matched with that in **(A)**. **(A–C)** patient samples are 50 total. **(D)** Kaplan-Meier survival analysis of all HCC patients (low NQO1, N=273; high NQO1, n=91) according to NQO1 mRNA expression in the TCGA database. Days to death: the number of days from the date of the initial pathological diagnosis to the date of death for the case in the investigation.

Our previous studies and other reports have shown that NQO1 enzyme levels were elevated, whereas catalase (gene: *CAT*) levels were lower in NSCLC and pancreatic cancers than in associated normal tissues, suggesting that the *NQO1*/*CAT* ratios in tumor tissue versus associated normal tissue are an important and highly exploitable therapeutic window (8, 11, 23). To investigate whether the *NQO1:CAT* ratios are a potential therapeutic window in liver cancer, we analyzed *NQO1* and *CAT* expression in liver hepatocellular carcinoma (LIHC) from The Cancer Genome Atlas (TCGA). Our analysis revealed that the mRNA levels of *NQO1* were significantly elevated (**Figure 1A**, *p* = 1.5×10^-7^), while *CAT* levels were notably lower (**Figure 1B**, *p* = 7.8×10^-8^) in tumor tissues than in matched normal tissues. Consistently, markedly higher *NQO1:CAT* ratios were observed in these tumor samples than in normal samples (**Figure 1C**, *p* = 1.5×10^-9^). Moreover, we also found that patients with high NQO1 expression had a significantly lower overall survival rate than those with low *NQO1* expression (**Figure 1D**, *p = 0.0075*). Together, these results indicate that the *NQO1:CAT* ratio is an ideal therapeutic window in liver cancer for *NQO1* bioactivatable drugs.

### Elevation of NQO1 Expression and Enzyme Activity in Hepatocellular Carcinoma Patients and Cell Lines

**Figure 2:**
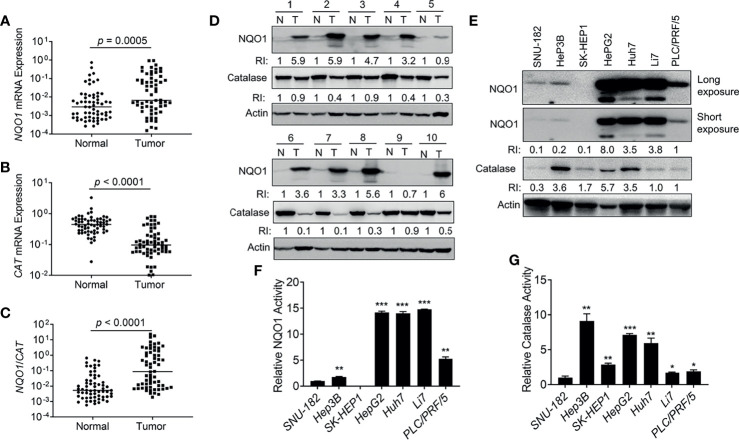
NQO1 and catalase expression in hepatocellular carcinoma patients and cell lines. **(A–C)** mRNA expression of *NQO1* **(A)**, *CAT* **(B)**, and *NQO1*/*CAT* Ratio **(C)** in 62 pairs of HCC patient tumor samples and associated normal tissues. **(D)** Representative western blotting analysis of NQO1 and catalase protein expression in HCC patient tumor samples and adjacent normal tissues. N, Normal; T, Tumor; RI, Relative Intensity. Data were measured as relative intensity (RI) of NQO1/Actin, and catalase/Actin. The protein expression in normal tissues was defined as 1. **(E)** NQO1 and catalase protein expression in HCC cell lines. Data were measured as RI of NQO1/Actin, and catalase/Actin. The protein expression in PLC/PRF/5 (last lane) was defined as 1. **(F, G)** Relative NQO1 and catalase enzyme activities in various HCC cell lines were detected by the NQO1 and catalase activity assay kit respectively, all error bars are means ± SDs. ***p < 0.001, **p < 0.01, *p < 0.05 (t tests).

To further confirm the above observations, we collected 62 pairs of clinical HCC patient samples and associated normal tissues to detect the mRNA levels of *NQO1* and *CAT*. Indeed, 69.4% (43/62) of HCC patient samples showed relatively higher *NQO1* mRNA levels than associated normal tissues (p = 0.0005, **Figure 2A**). In contrast, notably lower *CAT* mRNA levels (54/62 = 87.0%, *p* < 0.0001, **Figure 2B**) were observed in tumor tissues than in associated normal tissues. Concomitant high *NQO1* and low *CAT* mRNA levels (high NQO1:CAT ratios (**Figure 2C**, *p* < 0.0001) in HCC tumor tissue offer an ideal target for NQO1 bioactivatable drugs. Consistently, our western blotting analysis revealed that NQO1 protein levels were obviously elevated in 43.8% (28/64) of HCC tumor tissues, while catalase levels were repressed significantly in most HCC tumor tissues (**Figure 2D** and **Supplementary Figures S1A, B**). These results confirm our above observations that the relatively high *NQO1:CAT* ratios in HCC patients might be an exploitable therapeutic target in liver cancer. On the other hand, similar to the analysis in HCC patient samples, we observed that the liver cancer cell lines HepG2, Huh7 and Li7 showed high NQO1 levels; PLC/PRF/5 cells exhibited moderate NQO1 expression; and Hep3B, SNU-182, and SK-HEP1 cells expressed low or undetectable NQO1 (**Figure 2E**). Consistently, the NQO1 enzyme activity assay exhibited similar NQO1 activity in these cell lines (**Figure 2F**). Meanwhile, relatively high catalase protein levels accompanied by relative high catalase enzyme activities were observed in HepG2, Huh7 and Hep3B liver cancer cells (**Figures 2E, G**).

### Selective and Effective Killing of Hepatocellular Carcinoma Cells by β-Lapachone

**Figure 3:**
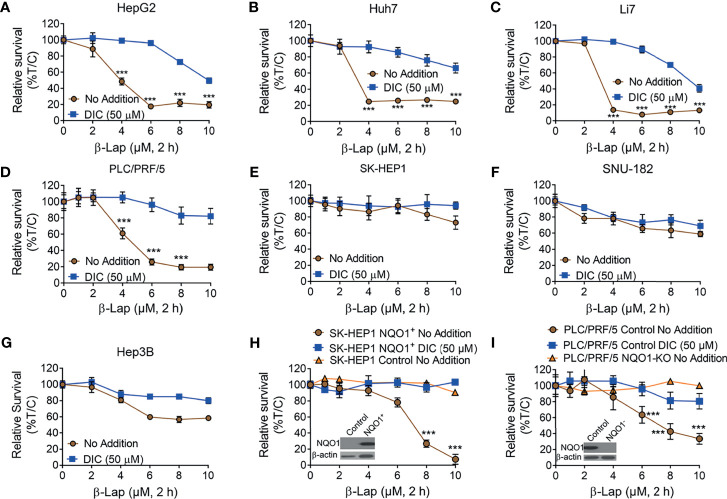
The cytotoxicity of β-Lapachone in hepatocellular carcinoma cells is NQO1-dependent. **(A–F)** HCC cells (HepG2 **(A)**, Huh7 **(B)**, Li7 **(C)**, PLC/PRF/5 **(D)**, SK-HEP1 **(E)**, SNU-182 **(F)**, and Hep3B **(G)**, were exposed to β-lap (0-10 µM), ± dicoumarol (DIC, 50 µM) for 2 h, and then relative survival was assessed. **(H, I)** NQO1-overexpressing SK-HEP1 cells **(H)** and stable NQO1 knockout PLC/PRF/5 cells **(I)** were treated as in **(A)**, and then cell viability was assessed. All error bars are means ± SDs. ***p < 0.001 (t tests). No addition, DMSO alone; DIC, dicoumarol; β-lap, β-Lapachone. Inset, NQO1 expression in NQO1-overexpressing SK-HEP1 and NQO1 knockout PLC/PRF/5 cells was confirmed by western blotting. %T/C, the mean of %Treated/Control.

It has been reported that NQO1 is a promising therapeutic target in multiple solid tumors, and the anticancer efficacy of β-lap is mainly mediated and promoted by NQO1 (7, 11, 17). Based on our above findings, we hypothesized that β-lap could effectively control cell growth in NQO1^+^ HCC cells. To this end, we treated HCC cells with β-lap or β-lap + dicoumarol (DIC, an NQO1-specific inhibitor). As expected, HepG2, Huh7, Li7 and PLC/PRF/5 cells, which endogenously express different levels of NQO1 (**Figure 2D**), showed significant sensitivity to β-lap (**Figures 3A–D**), while SK-HEP1, SNU-182 and Hep3B cells, which have undetectable or very low NQO1 expression, were resistant to β-lap exposure (**Figures 3E–G**). Next, we established stable NQO1-expressing SK-HEP1 cells and NOQ1 knockout PLC/PRF/5 cells to examine the lethality of β-lap. Consistently, SK-HEP1 cells were rendered hypersensitive to β-lap treatment after NQO1 expression, and DIC spared lethality (**Figure 3H**). Stable NQO1 knockout PLC/PRF/5 cells were much more resistant to β-lap than parental PLC/PRF/5 cells (**Figure 3I**). NQO1 expression in SK-HEP1 or knockout PLC/PRF/5 cells was confirmed by western blotting analysis (inset, **Figures 3H, I**). Together, these results demonstrate that β-lap efficiently kills HCC cells in an NQO1-mediated manner.

### β-Lapachone Induces NQO1-Dependent PARP1 Hyperactivation, ROS Formation and NAD^+^/ATP loss

**Figure 4:**
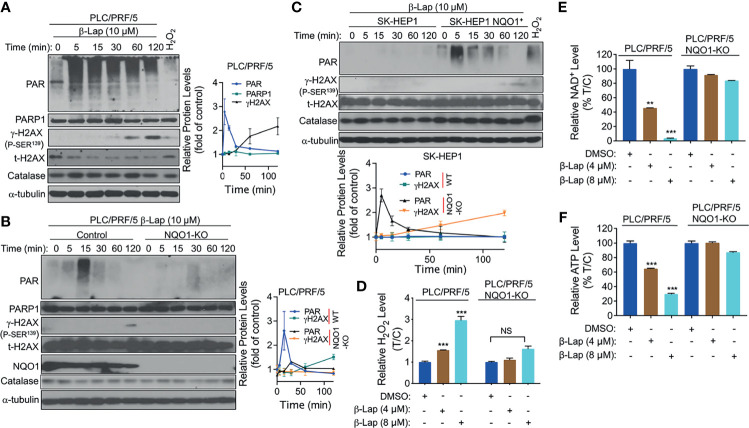
β-Lapachone induces NQO1-dependent PARP1 hyperactivation, ROS formation and NAD^+^/ATP loss. **(A–C)** PLC/PRF/5 **(A)**, NQO1 knockout PLC/PRF/5, **(B)** and NQO1-overexpressing SK-HEP1, (SK-HEP1 NQO1^+^) **(C)** cells were exposed to 10 µM β-lap for the indicated times. Cells were then harvested, and western blotting analysis was performed to detect the levels of PAR (PARP1 hyperactivation), PARP1, NQO1, catalase and γH2AX. The protein levels of PAR and γH2AX were quantified by Image J and normalized to α-tubulin, which was used as a loading control. **(D–F)** PLC/PRF/5 cells were treated with or without β-lap (4 or 8 µM) ± DIC (50 µM) for 2 h. Then, cells were subjected to measurement of H_2_O_2_ levels **(D)**, NAD^+^ levels **(E)** and ATP levels **(F)**. Data represent at least three independent sets of experiments. Error bars are means ± SDs. ***p < 0.001, **p < 0.01 (t tests). T/C, the mean of Treated/Control in **(D)** %T/C: the mean of %Treated/Control in **(E, F)**. NS, Not Statistically Significant.

Accumulating evidence suggests that exposure to β-lap causes DNA lesions in NQO1^+^ NSCLC, breast cancer, and pancreatic cancer cells, resulting in PARP hyperactivation in terms of the accumulation of poly(ADP-ribose)-PARP (PAR-PARP) posttranslational protein modification (11, 18, 24). To investigate whether PARP1 is involved in β-lap-induced cell death in HCC cells, we examined the effect of β-lap on poly(ADP-ribosyl)ated protein (PAR) accumulation, which is an indicator of PARP1 hyperactivation. First, β-lap-treated PLC/PRF/5 and Huh7 cells were analyzed for PAR formation using western blotting analysis. As shown, a lethal dose of β-lap (10 µM for PLC/PRF/5, 4 µM for Huh7 cells) significantly induced PARP1 hyperactivation, as indicated by a rapid rise in PAR formation and then an increase in DNA damage, as indicated by γH2AX expression over time (**Figure 4A** and **Supplementary Figure S2A**). To confirm that the β-lap-induced hyperactivation of PARP1 is NQO1-dependent, we next examined PAR formation in NQO1 knockout PLC/PRF/5 and NQO1-expressing SK-HEP1 cells. As expected, PAR formation was significantly inhibited by β-lap after NQO1 was stably knocked out in PLC/PRF/5 cells, accompanied by no detectable γH2AX expression (**Figure 4B**). A rapid increase and continuous level of PAR was detected after NQO1 re-expression in β-lap-resistant SK-HEP1 cells (**Figure 4C**). Consistently, DNA damage was observed in these SK-HEP1 cells. In addition, no significant change of catalase levels were noted in these β-lap-treated cells (**Figures 4A–C**). Taken together, these data indicate that β-lap induces PARP1 hyperactivation and DNA damage in NQO1^+^ HCC cells.

Intracellular ROS production is crucial for cancer cell death (25), and our previous studies show that NQO1 metabolizes β-lap in a futile redox cycle manner to generate ROS in other solid cancer cells (6, 26). Therefore, we investigated whether ROS are involved in β-lap-induced HCC cell death. We examined the levels of hydrogen peroxide (H_2_O_2_) as an indicator of intracellular ROS. After 2 h of exposure to a sublethal dose (4 or 8 µM) of β-lap, PLC/PRF/5 cells exhibited a significantly higher level of H_2_O_2_ (p < 0.001) than the untreated group (**Figure 4D**), while NQO1 KO cells showed no changes in H_2_O_2_ levels. Moreover, no obviously increase of H_2_O_2_ levels was observed in SK-HEP1 cells that have undetected NQO1 expression, while a significant increase of H_2_O_2_ levels was noted after reconstitution with NQO1 (**Supplementary Figure S2B**). Similarly, a significant increase in H_2_O_2_ levels in the β-lap-treated group was observed in NQO1^+^ Huh7 cells, and DIC blocked this increase (**Supplementary Figure S2C**). As reported previously by us and others (11, 27), β-lap-induced PAR-PARP1 formation consumes NAD^+^ and ATP, and together with our above results that PAR is induced rapidly and then decreases over time. Therefore, we examined NAD^+^ and ATP levels in β-lap-treated HCC cells. As shown in **Figures 4E, F** and **Supplementary Figures S2D, E**, dramatic NAD^+^ and ATP depletion was observed after NQO1^+^ cells were exposed to β-lap for 2 h. All these depletions were blocked in the NQO1 KO cells or DIC-treated group. When NQO1 expression was restored in SK-HEP1 cells, markedly increase of NAD^+^ and ATP levels were observed after treatment with β-lap (**Supplementary Figures S2F, G**). Together, these results demonstrate that β-lap induces cell stress in HCC cells by generating ROS, and disrupts essential metabolic nucleotides.

### β-Lapachone Causes Dramatic NQO1-dependent Total DNA Damage and Double-Strand Breaks in Hepatocellular Carcinoma Cells

**Figure 5:**
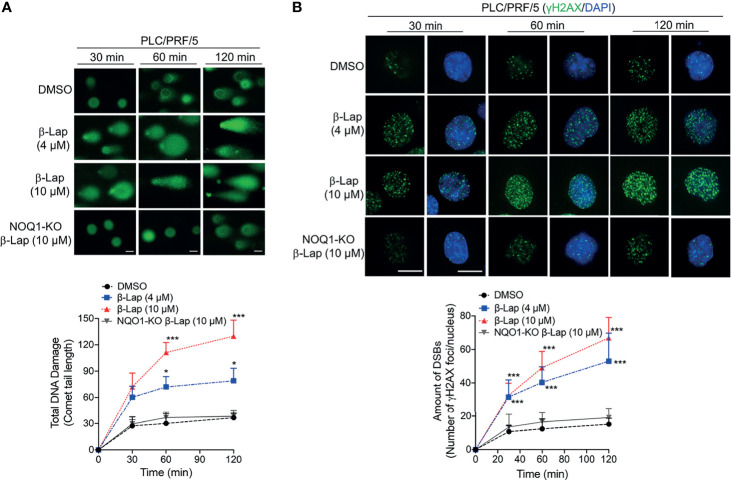
β-Lapachone induces NQO1-dependent DSB formation in hepatocellular carcinoma cells. Wild-type or NQO1 knockout PLC/PRF/5 cells were treated with a sublethal (4 µM) or lethal (10 µM) dose of β-lap for the indicated time (min), and then total DNA lesions were assessed using the alkaline comet assay. **(A)** Comet tail lengths were imaged under an immunofluorescence microscope. **(B)** DSBs were quantified by γH2AX foci/nuclei. Data represent the means ± SDs. Student’s t tests were performed. Scale bar = 10 µm, ***p < 0.001, *p < 0.05.

According to previous reports, ROS release can mediate and promote DNA damage (28, 29), and our above results show that β-lap generates ROS in HCC cells. Therefore, we investigated total DNA damage under β-lap treatment *via* an alkaline comet assay in PLC/PRF/5 cells. In NQO1-expressing PLC/PRF/5 wild-type cells, a sublethal dose of β-lap (4 μM) caused total DNA damage, as indicated by the comet tail, as early as 30 min, and a lethal dose of β-lap (10 μM) markedly increased DNA damage (**Figure 5A**). In contrast, when *NQO1* was knocked out, no obvious DNA tails were observed even at a lethal dose of β-lap (10 μM) (**Figure 5A**). Consistently, quantification of the comet tail length confirmed these observations. On the other hand, our previous study suggested that when PARP hyperactivity is exhausted, cells attempt to replicate despite the damage to AP sites or the presence of SSBs, and the damage becomes hypersensitive to the oxidative stress caused by β-lap and then induces DSBs (21). In fact, we observed γH2AX expression after β-lap treatment in NQO1^+^ HCC cells, especially when PAR levels were exhausted (**Figure 4**). To further confirm whether β-lap induces DSBs in HCC cells, we examined γH2AX expression *via* immunofluorescence staining. As shown in **Figure 5B**, dramatic increases in DNA DSB formation were noted in the PLC/PRF/5 cells that were treated with a sublethal or lethal dose of β-lap compared to the untreated cells as early as 30 min. Even at a lethal dose of β-lap (10 µM), NQO1 knockout cells showed no obvious increase in γH2AX foci. The observations were confirmed by the quantification of γH2AX foci formation (**Figure 5B**). Taken together, these data suggest that exposure of NQO1^+^ HCC cells to β-lap results in cell death due to significant DNA DSBs.

### β-Lapachone Significantly Suppresses Tumor Growth and Prolongs Survival in HCC Mouse Models

**Figure 6:**
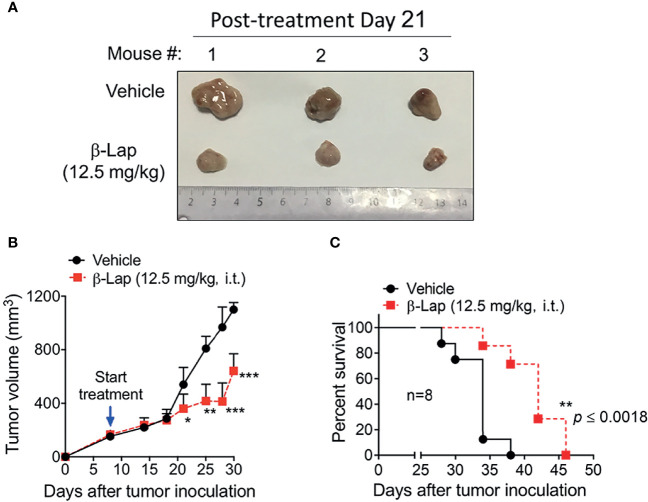
Antitumor efficacy of β-Lapachone in a mouse xenograft model. The subcutaneous xenograft tumor model was established by injection of 5×10^6^ PLC/PRF/5 cells into male NOD/SCID mice. After 8 days, mice were treated with vehicle (HPβCD) or HPβCD-β-Lap (12.5 mg/kg) by intratumor injections every other day for five injections. **(A)** Representative tumors at day 21 post-treatment. **(B)** Tumor volume at the indicated time (days). **(C)** Kaplan-Meier survival curves. ***p < 0.001, **p < 0.01, *p < 0.05.

Relatively high NQO1 and low catalase expression in various HCC patients and cell lines indicates a potent therapeutic window in liver carcinoma for β-lap, and our above data confirmed that β-lap efficiently represses tumor cell growth *in vitro*. To address the antitumor efficacy of β-lap *in vivo*, we established xenograft liver cancer models. First, 5×10^6^ PLC/PRF/5 cells/mouse were implanted subcutaneously into NOD/SCID mice. When the tumor size reached 150 mm^3^, the mice were grouped and treated with vehicle (HPβCD, intratumor (i.t.)) or HPβCD-β-lap (hereafter referred to as β-lap, at 12.5 mg/kg, i.t.) every other day for a total of five injections. Tumor volume and survival were scored to monitor tumor growth and response (**Figures 6A–C**). Exposure to β-lap resulted in a dramatic decrease in tumor growth compared with vehicle group mice, confirmed by the quantification of tumor volume (**Figures 6A, B**). Moreover, overall survival also showed significant antitumor efficacy of β-lap (**Figure 6C**). This model suggests that β-lap is a good candidate agent to kill NQO1^+^ HCC *in vivo.*

## Discussion

Here, we show a potential antitumor effect of β-lap in HCC and reveal that β-lap efficiently killed NQO1-overexpressing HCC cells without affecting NQO1-low-expressing cells or tissues. As the most common type of primary liver cancer, HCC often occurs in people with long-term liver diseases and is generally diagnosed with advanced disease, leading to systemic therapy (30, 31). At present, there are only a few efficacious drugs for HCC treatment or the drugs cause severe side effects (32, 33). Thus, exploiting new drugs and identifying differences between carcinomas and healthy tissue are critical for HCC treatment. Previous reports suggest that β-lap is a competent tumor-selective agent against many NQO1^+^ solid cancers, such as NSCLC, pancreatic and breast cancers (6, 11, 34–36), while the antitumor effect of this drug in liver cancer is still unknown. Except the expression and activity of NQO1, the cytotoxicity of β-lap is also driven by ROS-metabolizing enzymes catalase and SOD1 expression and activity. catalase is an important resistance factor in β-lap-induced cytotoxicity and this resistance could be enhanced by superoxide dismutase (SOD) (37). The *NQO1:CAT* ratios are suggested to be a therapeutic window in NSCLC, pancreatic and breast cancers (11, 18). In our study, analysis of 64 clinical patient samples of HCC revealed that 43.8% of patients exhibited NQO1 overexpression, and the majority of patients showed low CAT levels in tumors compared with adjacent normal tissues. Moreover, LIHC data from TCGA demonstrated that patient overall survival significantly correlated with *NQO1* expression. These results suggest that relatively high NQO1:CAT ratios in tumor tissues could provide a therapeutic window for using NQO1 bioactivatable drugs such as β-lap to kill HCC. In fact, cell viability showed that β-lap efficiently killed PLC/PRF/5, Huh7, and Li7 cells, which have high NQO1 expression, but did not affect NOQ1^-^ cell growth (**Figure 2**).

β-lap was reported to induce cancer cell death *via* an NQO1-dependent programmed necrotic pathway, which caused robust ROS elevation and PARP1 hyperactivation (6, 38). In HCC cells, we observed β-lap-induced NQO1-selective elevated H_2_O_2_ and a rapid and transient increase in PAR formation, followed by DNA damage over time. On the other hand, PARP1 uses NAD^+^ as a substrate to perform PAR posttranslational modification of proteins, resulting in NAD^+^ and ATP losses (27, 39). Our β-lap-treated HCC cells indeed exhibited NQO1-dependent NAD^+^ and ATP depletion. Collectively, our investigation of clinical patient samples of HCC and *in vitro* results offer a therapeutic window and potential use of β-lap in HCC.

β-lap has an apparently broader NQO1-dependent therapeutic window in HCC. NQO1 is overexpressed in numerous human cancers, and our previous studies together with others demonstrate that β-lap has efficacy in tumor-selective cell growth control and produced promising preclinical results (6, 11, 19, 34, 36). Our preclinical model exhibited an efficient antitumor effect of β-lap in HCC in which β-lap-treated mice had dramatically decreased tumor growth and prolonged survival compared to control mice. Together with our *in vitro* results, we anticipate that β-lap would be an extremely efficacious tumor-selective therapy against HCC and other kinds of liver cancer. Furthermore, the data presented in this study reveal that NQO1 and the *NQO1:CAT* ratio could be used as biomarkers to examine the efficacy of NQO1 bioactivatable drugs in HCC or other kinds of liver cancers. In addition, our *in vitro* and *in vivo* data could translate our findings regarding β-lap in HCC to the clinic. Moreover, because β-lap alone induces tumor programmed necrotic cell death that could induce many cytokines or other side effects *in vivo*, our recent report revealed that low-dose β-lap combined with a PARP inhibitor switched the pathway to apoptosis (11), which implies that combination therapy between β-lap and other clinical drugs would be worth exploring. Finally, immunotherapy with immune checkpoint inhibitors such as PD-1 inhibitors has shown promise in HCC (40). Clinical data showed that only approximately 15-20% of HCC patients exhibited a response, and a fraction of HCC patients could benefit from this therapy (41, 42). We recently revealed that β-lap not only directly kills tumor cells but also increases tumor immunogenicity by triggering immunogenic cell death and overcoming immunotherapy resistance (43). Thus, we propose that β-lap could exert a synergistic effect with immune checkpoint inhibitors and enhance the antitumor immune response in HCC.

Taken together, our study demonstrated that β-lap, a novel NQO1 bioactivatable drug, selectively kills HCC cells expressing NQO1 through inducing ROS and PAR formation, NAD^+^ and ATP depletion and lethal DNA damage. High *NQO1:CAT* ratios in HCC tumors but low ratios in normal tissues offer an optimal therapeutic window and an ideal therapeutic target for β-lap.

## Data Availability Statement

The original contributions presented in the study are included in the article/**Supplementary Material**. Further inquiries can be directed to the corresponding author.

## Ethics Statement

The studies involving human participants were reviewed and approved by Zhongshan Hospital of Xiamen University. The patients/participants provided their written informed consent to participate in this study. The animal study was reviewed and approved by the IU IACUC committee.

## Author Contributions

LJ, WZ, and XH designed the experiments, analyzed the data, and wrote the manuscript. XH supervised the project. SL, YY, HZ, and JW. analyzed the bioinformatic data. TF, FF, YL, YZ, XS, and JWW provided help with the experiments. LJ, WZ, YC, JW, and XH reviewed and edited the manuscript. All authors contributed to the article and approved the submitted version.

## Funding

This work was supported by NIH R01 grants CA221158, CA224493 and CA240952 to XH. This work was also supported by the IU Simon Comprehensive Cancer Center (Grant P30CA082709), the Purdue University Center for Cancer Research (Grant P30CA023168) and the Walther Cancer Foundation.

## Conflict of Interest

The authors declare that the research was conducted in the absence of any commercial or financial relationships that could be construed as a potential conflict of interest.

## Publisher’s Note

All claims expressed in this article are solely those of the authors and do not necessarily represent those of their affiliated organizations, or those of the publisher, the editors and the reviewers. Any product that may be evaluated in this article, or claim that may be made by its manufacturer, is not guaranteed or endorsed by the publisher.

## Supplementary Material

Supplementary Figure S1 NQO1 and catalase expression in hepatocellular carcinoma patients. **(A, B)** Western blotting analysis of NQO1 and catalase protein expressions in 54 pairs of HCC patient tumor samples and adjacent normal tissues. N, Normal; T, Tumor.

Supplementary Figure S2 β-Lapachone induces NQO1-dependent PARP1 hyperactivation, ROS formation and NAD^+^/ATP loss in Huh7 cells. **(A)** Huh7 cells were exposed to 4 µM β-lap at the indicated time, then cells were harvested and western blotting analysis was to detect the levels of PAR (PARP1 hyperactivation) and γH2AX. **(B–G)** SK-HEP1, SK-HEP1 NQO1^+^, and Huh7 cells were treated with or without β-lap ± DIC (50 µM) for 2 h. Then cells were measured for H_2_O_2_ levels **(B, C)**, NAD^+^ levels **(D, F)**, and ATP levels **(E, G)**. Data represent at least three independent sets of experiments. Error bars are means ± SD. ***p < 0.001, **p < 0.01, *p < 0.05 (t tests).

## Abbreviations

HCC, hepatocellular carcinoma; β-lap, β-Lapachone; NQO1, NADPH:quinone oxidoreductase 1; *CAT*, catalase; TCGA, The Cancer Genome Atlas; DSB, double-strand break; ROS, reactive oxygen species; NSCLC, non-small-cell lung cancer; H_2_O_2_, Hydrogen peroxide; DIC, dicoumarol; PAR, poly(ADP-ribosyl)ated protein; NOD/SCID, nonobese diabetic/severe combined immunodeficiency; LIHC, liver hepatocellular carcinoma.

## References

[1] Llovet JM Zucman-Rossi J Pikarsky E Sangro B Schwartz M Sherman M. Hepatocellular Carcinoma. Nat Rev Dis Primers (2016) 2:16018. doi: 10.1038/nrdp.2016.18 27158749

[2] Marrero JA Kulik LM Sirlin CB Zhu AX Finn RS Abecassis MM. Diagnosis, Staging, and Management of Hepatocellular Carcinoma: 2018 Practice Guidance by the American Association for the Study of Liver Diseases. Hepatology (2018) 68:723–50. doi: 10.1002/hep.29913 29624699

[3] Lee SH Song IH Noh R Kang HY Kim SB Ko SY. Clinical Outcomes of Patients With Advanced Hepatocellular Carcinoma Treated With Sorafenib: A Retrospective Study of Routine Clinical Practice in Multi-Institutions. BMC Cancer (2015) 15:236. doi: 10.1186/s12885-015-1273-2 25885683 PMC4403976

[4] Siegel D Ross D. Immunodetection of NAD(P)H:quinone Oxidoreductase 1 (NQO1) in Human Tissues. Free Radical Biol Med (2000) 29:246–53. doi: 10.1016/S0891-5849(00)00310-5 11035253

[5] Zhang K Chen D Ma K Wu X Hao H Jiang S. NAD(P)H:Quinone Oxidoreductase 1 (NQO1) as a Therapeutic and Diagnostic Target in Cancer. J Medicinal Chem (2018) 61:6983–7003. doi: 10.1021/acs.jmedchem.8b00124 29712428

[6] Bey EA Bentle MS Reinicke KE Dong Y Yang CR Girard L. An NQO1- and PARP-1-Mediated Cell Death Pathway Induced in Non-Small-Cell Lung Cancer Cells by Beta-Lapachone. Proc Natl Acad Sci USA (2007) 1041:1832–7. doi: 10.1073/pnas.0702176104 PMC1913860 17609380

[7] Cao L Li LS Spruell C Xiao L Chakrabarti G Bey EA. Tumor-Selective, Futile Redox Cycle-Induced Bystander Effects Elicited by NQO1 Bioactivatable Radiosensitizing Drugs in Triple-Negative Breast Cancers. Antioxid Redox Signaling (2014) 21:237–50. doi: 10.1089/ars.2013.5462 PMC4060774 24512128

[8] Chakrabarti G Silvers MA Ilcheva M Liu Y Moore ZR Luo X. Tumor-Selective Use of DNA Base Excision Repair Inhibition in Pancreatic Cancer Using the NQO1 Bioactivatable Drug, Beta-Lapachone. Sci Rep (2015) 5:17066. doi: 10.1038/srep17066 26602448 PMC4658501

[9] Dong Y Chin SF Blanco E Bey EA Kabbani W Xie XJ. Intratumoral Delivery of Beta-Lapachone *via* Polymer Implants for Prostate Cancer Therapy. Clin Cancer Res: an Off J Am Assoc Cancer Res (2009) 15:131–9. doi: 10.1158/1078-0432.CCR-08-1691 PMC2845536 19118040

[10] Dong Y Bey EA Li LS Kabbani W Yan J Xie XJ. Prostate Cancer Radiosensitization Through Poly(ADP-Ribose) Polymerase-1 Hyperactivation. Cancer Res (2010) 70:8088–96. doi: 10.1158/0008-5472.CAN-10-1418 PMC2955807 20940411

[11] Huang X Motea EA Moore ZR Yao J Dong Y Chakrabarti G. Leveraging an NQO1 Bioactivatable Drug for Tumor-Selective Use of Poly(ADP-Ribose) Polymerase Inhibitors. Cancer Cell (2016) 30:940–52. doi: 10.1016/j.ccell.2016.11.006 PMC5161231 27960087

[12] Cheng ML Lu YF Chen H Shen ZY Liu J. Liver Expression of Nrf2-Related Genes in Different Liver Diseases. Hepatobiliary Pancreatic Dis International: HBPD Int (2015) 14:485–91. doi: 10.1016/S1499-3872(15)60425-8 26459724

[13] Lin L Sun J Tan Y Li Z Kong F Shen Y. Prognostic Implication of NQO1 Overexpression in Hepatocellular Carcinoma. Hum Pathol (2017) 69:31–7. doi: 10.1016/j.humpath.2017.09.002 28964792

[14] Li WY Zhou HZ Chen Y Cai XF Tang H Ren JH. NAD(P)H: Quinone Oxidoreductase 1 Overexpression in Hepatocellular Carcinoma Potentiates Apoptosis Evasion Through Regulating Stabilization of X-Linked Inhibitor of Apoptosis Protein. Cancer Lett (2019) 451:156–67. doi: 10.1016/j.canlet.2019.02.053 30867140

[15] Zhou HZ Zeng HQ Yuan D Ren JH Cheng ST Yu HB. NQO1 Potentiates Apoptosis Evasion and Upregulates XIAP *via* Inhibiting Proteasome-Mediated Degradation SIRT6 in Hepatocellular Carcinoma. Cell Communication Signal: CCS (2019) 17:168. doi: 10.1186/s12964-019-0491-7 PMC6915971 31842909

[16] Planchon SM Wuerzberger S Frydman B Witiak DT Hutson P Church DR. Beta-Lapachone-Mediated Apoptosis in Human Promyelocytic Leukemia (HL-60) and Human Prostate Cancer Cells: A P53-Independent Response. Cancer Res (1995) 55:3706–11. PMC4807624 7641180

[17] Chakrabarti G Moore ZR Luo X Ilcheva M Ali A Padanad M. Targeting Glutamine Metabolism Sensitizes Pancreatic Cancer to PARP-Driven Metabolic Catastrophe Induced by ss-Lapachone. Cancer Metab (2015) 3:12. doi: 10.1186/s40170-015-0137-1 26462257 PMC4601138

[18] Bey EA Reinicke KE Srougi MC Varnes M Anderson VE Pink JJ. Catalase Abrogates Beta-Lapachone-Induced PARP1 Hyperactivation-Directed Programmed Necrosis in NQO1-Positive Breast Cancers. Mol Cancer Ther (2013) 12:2110–20. doi: 10.1158/1535-7163.MCT-12-0962 PMC3807805 23883585

[19] Beg MS Huang X Silvers MA Gerber DE Bolluyt J Sarode V. Using a Novel NQO1 Bioactivatable Drug, Beta-Lapachone (ARQ761), to Enhance Chemotherapeutic Effects by Metabolic Modulation in Pancreatic Cancer. J Surg Oncol (2017) 116:83–8. doi: 10.1002/jso.24624 PMC5509448 28346693

[20] Huang X Dong Y Bey EA Kilgore JA Bair JS Li LS. An NQO1 Substrate With Potent Antitumor Activity That Selectively Kills by PARP1-Induced Programmed Necrosis. Cancer Res (2012) 72:3038–47. doi: 10.1158/0008-5472.CAN-11-3135 PMC4795165 22532167

[21] Motea EA Huang X Singh N Kilgore JA Williams NS Xie XJ. NQO1-Dependent, Tumor-Selective Radiosensitization of Non-Small Cell Lung Cancers. Clin Cancer Res: an Off J Am Assoc Cancer Res (2019) 25:2601–9. doi: 10.1158/1078-0432.CCR-18-2560 PMC6788754 30617135

[22] Zhao B Zhao W Wang Y Xu Y Xu J Tang K. Connexin32 Regulates Hepatoma Cell Metastasis and Proliferation *via* the P53 and Akt Pathways. Oncotarget (2015) 6:10116–33. doi: 10.18632/oncotarget.2687 PMC4496344 25426556

[23] Li Z Zhang Y Jin T Men J Lin Z Qi P. NQO1 Protein Expression Predicts Poor Prognosis of Non-Small Cell Lung Cancers. BMC Cancer (2015) 15:207. doi: 10.1186/s12885-015-1227-8 25880877 PMC4396547

[24] Zhang F Xie R Munoz FM Lau SS Monks TJ. PARP-1 Hyperactivation and Reciprocal Elevations in Intracellular Ca2+ During ROS-Induced Nonapoptotic Cell Death. Toxicol Sci: An Off J Soc Toxicol (2014) 140:118–34. doi: 10.1093/toxsci/kfu073 PMC4081636 24752504

[25] Zou Z Chang H Li H Wang S. Induction of Reactive Oxygen Species: An Emerging Approach for Cancer Therapy. Apoptosis: An Int J Programmed Cell Death (2017) 22:1321–35. doi: 10.1007/s10495-017-1424-9 28936716

[26] Li LS Reddy S Lin ZH Liu S Park H Chun SG. NQO1-Mediated Tumor-Selective Lethality and Radiosensitization for Head and Neck Cancer. Mol Cancer Ther (2016) 15:1757–67. doi: 10.1158/1535-7163.MCT-15-0765 PMC5123441 27196777

[27] Pieper AA Blackshaw S Clements EE Brat DJ Krug DK White AJ. Poly(ADP-Ribosyl)Ation Basally Activated by DNA Strand Breaks Reflects Glutamate-Nitric Oxide Neurotransmission. Proc Natl Acad Sci USA (2000) 97:1845–50. doi: 10.1073/pnas.97.4.1845 PMC26524 10677544

[28] Moloney JN Cotter TG. ROS Signalling in the Biology of Cancer. Semin Cell Dev Biol (2018) 80:50–64. doi: 10.1016/j.semcdb.2017.05.023 28587975

[29] Srinivas US Tan BWQ Vellayappan BA Jeyasekharan AD. ROS and the DNA Damage Response in Cancer. Redox Biol (2019) 25:101084. doi: 10.1016/j.redox.2018.101084 30612957 PMC6859528

[30] Yu SJ. A Concise Review of Updated Guidelines Regarding the Management of Hepatocellular Carcinoma Around the World: 2010-2016. Clin Mol Hepatol (2016) 22:7–17. doi: 10.3350/cmh.2016.22.1.7 27044761 PMC4825164

[31] Qadan M Kothary N Sangro B Palta M. The Treatment of Hepatocellular Carcinoma With Portal Vein Tumor Thrombosis. Am Soc Clin Oncol Educ book Am Soc Clin Oncol Annu Meeting (2020) 40:1–8. doi: 10.1200/EDBK_280811 32213090

[32] Nie J Lin B Zhou M Wu L Zheng T. Role of Ferroptosis in Hepatocellular Carcinoma. J Cancer Res Clin Oncol (2018) 144:2329–37. doi: 10.1007/s00432-018-2740-3 PMC11813439 30167889

[33] Al-Salama ZT Syed YY Scott LJ. Lenvatinib: A Review in Hepatocellular Carcinoma. Drugs (2019) 79:665–74. doi: 10.1007/s40265-019-01116-x 30993651

[34] Li LS Bey EA Dong Y Meng J Patra B Yan J. Modulating Endogenous NQO1 Levels Identifies Key Regulatory Mechanisms of Action of Beta-Lapachone for Pancreatic Cancer Therapy. Clin Cancer Res: an Off J Am Assoc Cancer Res (2011) 17:275–85. doi: 10.1158/1078-0432.CCR-10-1983 PMC4806682 21224367

[35] Silvers MA Deja S Singh N Egnatchik RA Sudderth J Luo X. The NQO1 Bioactivatable Drug, Beta-Lapachone, Alters the Redox State of NQO1+ Pancreatic Cancer Cells, Causing Perturbation in Central Carbon Metabolism. J Biol Chem (2017) 292:18203–16. doi: 10.1074/jbc.M117.813923 PMC5672043 28916726

[36] Yang Y Zhou X Xu M Piao J Zhang Y Lin Z. Beta-Lapachone Suppresses Tumour Progression by Inhibiting Epithelial-to-Mesenchymal Transition in NQO1-Positive Breast Cancers. Sci Rep (2017) 7:2681. doi: 10.1038/s41598-017-02937-0 28578385 PMC5457413

[37] Torrente L Prieto-Farigua N Falzone A Elkins CM Boothman DA Haura EB. Inhibition of TXNRD or SOD1 Overcomes NRF2-Mediated Resistance to Beta-Lapachone. Redox Biol (2020) 30:101440. doi: 10.1016/j.redox.2020.101440 32007910 PMC6997906

[38] Pink JJ Planchon SM Tagliarino C Varnes ME Siegel D Boothman DA. NAD(P)H:Quinone Oxidoreductase Activity Is the Principal Determinant of Beta-Lapachone Cytotoxicity. J Biol Chem (2000) 275:5416–24. doi: 10.1074/jbc.275.8.5416 10681517

[39] Kim MY Zhang T Kraus WL. Poly(ADP-Ribosyl)Ation by PARP-1: ‘PAR-Laying’ NAD+ Into a Nuclear Signal. Genes Dev (2005) 19:1951–67. doi: 10.1101/gad.1331805 16140981

[40] Sia D Jiao Y Martinez-Quetglas I Kuchuk O Villacorta-Martin C Castro de Moura M. Identification of an Immune-Specific Class of Hepatocellular Carcinoma, Based on Molecular Features. Gastroenterology (2017) 153:812–26. doi: 10.1053/j.gastro.2017.06.007 PMC12166766 28624577

[41] Wehrenberg-Klee E Goyal L Dugan M Zhu AX Ganguli S. Y-90 Radioembolization Combined With a PD-1 Inhibitor for Advanced Hepatocellular Carcinoma. Cardiovasc Interventional Radiol (2018) 41:1799–802. doi: 10.1007/s00270-018-1993-1 29845347

[42] Zhu AX Finn RS Edeline J Cattan S Ogasawara S Palmer D. Pembrolizumab in Patients With Advanced Hepatocellular Carcinoma Previously Treated With Sorafenib (KEYNOTE-224): A Non-Randomised, Open-Label Phase 2 Trial. Lancet Oncol (2018) 19:940–52. doi: 10.1016/S1470-2045(18)30351-6 29875066

[43] Li X Liu Z Zhang A Han C Shen A Jiang L. NQO1 Targeting Prodrug Triggers Innate Sensing to Overcome Checkpoint Blockade Resistance. Nat Commun (2019) 10:3251. doi: 10.1038/s41467-019-11238-1 31324798 PMC6642086

