# Supplementary figures and images for: β-Lapachone Selectively Kills Hepatocellular Carcinoma Cells by Targeting NQO1 to Induce Extensive DNA Damage and PARP1 Hyperactivation
Source: Front Oncol. 2021 Oct 5;11:747282. doi: 10.3389/fonc.2021.747282 (PMC8523939; doi:10.3389/fonc.2021.747282)

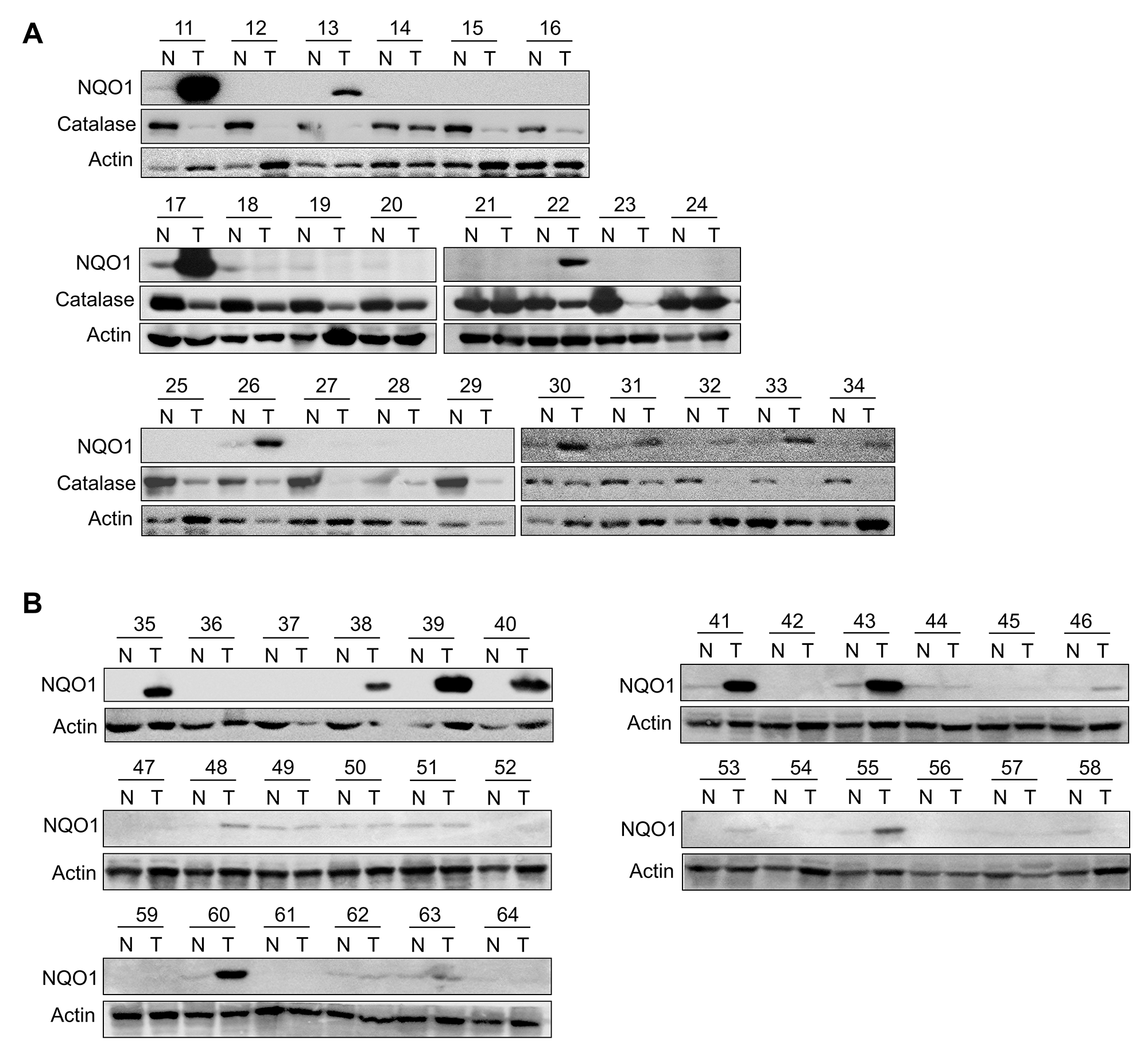

Supplement: Supplementary Figure S1 — NQO1 and catalase expression in hepatocellular carcinoma patients. (A, B) Western blotting analysis of NQO1 and catalase protein expressions in 54 pairs of HCC patient tumor samples and adjacent normal tissues. N, Normal; T, Tumor. [file Image_1.tif]

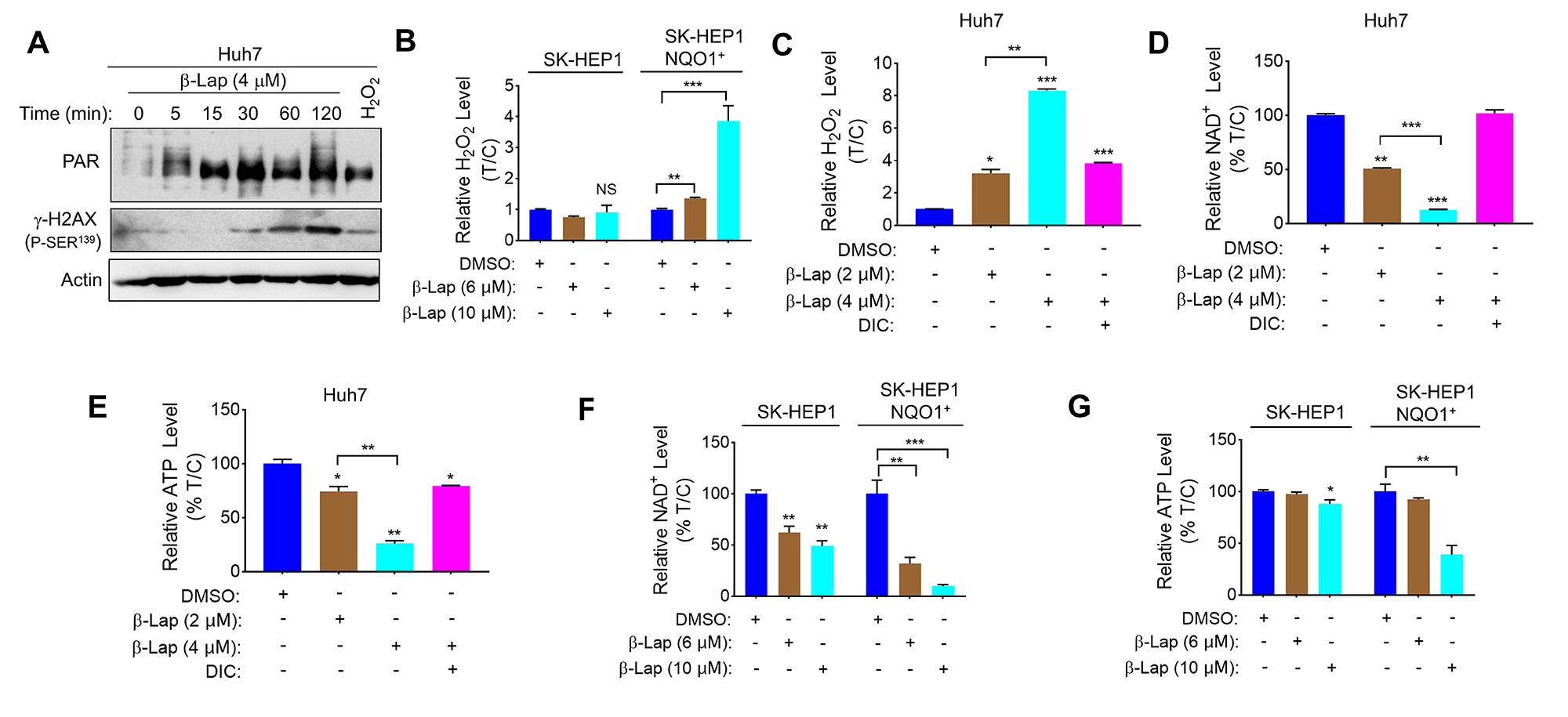

Supplement: Supplementary Figure S2 — β-Lapachone induces NQO1-dependent PARP1 hyperactivation, ROS formation and NAD+/ATP loss in Huh7 cells. (A) Huh7 cells were exposed to 4 µM β-lap at the indicated time, then cells were harvested and western blotting analysis was to detect the levels of PAR (PARP1 hyperactivation) and γH2AX. (B–G) SK-HEP1, SK-HEP1 NQO1+, and Huh7 cells were treated with or without β-lap ± DIC (50 µM) for 2 h. Then cells were measured for H2O2 levels (B, C), NAD+ levels (D, F), and ATP levels (E, G). Data represent at least three independent sets of experiments. Error bars are means ± SD. ***p < 0.001, **p < 0.01, *p < 0.05 (t tests). [file Image_2.tif]
